# Feature attention and accent recognition: human listeners’ responses to five Northern English accents

**DOI:** 10.3389/fpsyg.2025.1613018

**Published:** 2025-12-04

**Authors:** Chris Montgomery, Hielke Vriesendorp, Gareth Walker

**Affiliations:** 1 School of English, University of Sheffield, Sheffield, United Kingdom; 2 Department of Languages, Literature and Communication, Utrecht University, Utrecht, Netherlands

**Keywords:** accent perception, linguistic salience, regional variation, sociophonetics, speech processing

## Abstract

This study investigates how human listeners perceive and locate Northern English accents, with a focus on the linguistic features that attract attention during accent recognition. Although sociolinguistic research often centers on specific phonetic variables, it is unclear whether these align with the cues non-linguists naturally notice when identifying regional varieties. To address this, we used a real-time linguistic attention method to examine which features listeners attended to as they attempted to identify the origin of speakers from five Northern English cities: Leeds, Liverpool, Manchester, Newcastle, and Sheffield. Crucially, listeners were not directed to focus on any particular features. Data from 98 participants revealed substantial variation in recognition accuracy. While Liverpool and Newcastle voices were frequently identified correctly, accents from Leeds, Manchester, and Sheffield proved more difficult to place. Participants consistently attended to salient features such as the bath and strut vowels, along with more locally specific features like fricated /k/ in Liverpool and glottalized /t/ in Newcastle. Accents with multiple distinct cues were more reliably identified, suggesting that a cluster of reinforcing features enhances perceptual success. The findings show that listener attention is guided by both cognitive and social salience, and that listeners rely on a broader and more socially grounded set of features than those often prioritized in computational models of accent classification. By revealing which features listeners attend to spontaneously, this study contributes to understanding the cognitive processes underpinning regional accent perception and offers new insights into the interplay between linguistic variation, salience, and social indexing.

## 1 Introduction

Most sociolinguistic work on phonetic variation focuses on a selection of features or a singular feature which is deemed relevant and distinctive for the variety in which it occurs. The selection of these features or this feature is generally made (implicitly) by the researcher. Yet, it is not a given that what is distinctive or salient to a researcher is also distinctive or salient to other language users. A recent study by Strycharczuk et al. (2020) used machine learning to identify which vowel features most strongly differentiated five Northern English cities. Their random-forest models highlighted cues not widely studied in sociolinguistics, raising the question of whether these ‘algorithmically salient’ features are also those that human listeners notice when identifying accents.

This is the premise of the present study. Using the Salient Language in Context (SLIC) method (Montgomery et al., 2025), which records listener attention to specific features in real time, we directly test whether human listeners attend to the same features identified by Strycharczuk et al. We examine perceptions of five speakers from the same Northern cities, thereby providing a human counterpoint to the machine classification results.

In this article we address two research questions: (i) how accurate are listeners at placing voice samples from five Northern English cities? and (ii) what features do listeners attend to as they attempt to place speakers from the five cities? Over the course of the paper, we demonstrate the link between feature attention and different types of salience. We find a variable ability amongst listeners to locate speakers of different Northern English varieties and show how specific features present in speaker guises can affect listener perceptions of speaker provenance. We are able to corroborate that the features human participants use to distinguish between the five cities are much more in line with those features most often studied by linguists than the random-forest models reported in Strycharczuk et al. (2020). Our findings therefore reveal that listeners’ attention patterns do not mirror the algorithmic predictions but instead align more closely with the features traditionally studied in sociolinguistics. Furthermore, over the course of the paper we present evidence that allows us to argue that social salience should be considered gradable in the same way as cognitive salience.

The work presented in this paper is original, presenting newly collected data that aims to ask questions about how listeners are able to locate speakers. Crucially we are also able to analyze the features that listeners identify as they attempt to place speakers. The research is significant as it shows that it is possible to document listener attention to linguistic features in a meaningful way and addresses how this attention relates to sample placements for the first time. The research was undertaken using a large sample of listeners, using coding and data processing conforming to best-practice guidelines, and our analysis draws on current thinking about salience, attention, and sociolinguistic perception, all of which testifies to its rigor.

### 1.1 Human accent recognition

Human accent recognition research has tended to use two main types of approach: forced- and free-choice tasks. Forced-choice methods request that listeners determine where they think a speaker is from based on a list of predetermined locations (e.g., Bush, 1967; van Bezooijen and Gooskens, 1999; Plichta and Preston, 2005; Clopper and Pisoni, 2006; Hanani et al., 2013; Ruch, 2018). These approaches may provide a list of towns or cities (e.g., Plichta and Preston, 2005; Hanani et al., 2013) or visual display of regions (e.g., Clopper and Pisoni, 2006) and require listeners to allocate a sample to one of them, or to choose one of two locations for a given stimulus (see Ruch, 2018).

Free-choice methods use a range of approaches, including simply asking respondents where they think a speaker is from (Dragojevic et al., 2018, p. 36; Braber et al., 2022), requesting listeners to indicate on a map where they believe a speaker to come from (e.g., Montgomery, 2011; Cole, 2021; Pinget and Voeten, 2023), or requesting that listeners to group voice samples according to perceptual similarity (see Clopper and Pisoni, 2007; Clopper and Bradlow, 2009; Bent et al., 2016). Some approaches also ask for listeners’ reasons for their location choice after samples have been played (Tomé Lourido and Evans, 2021; Braber et al., 2022).

In this research we employ a forced-choice method involving listeners allocating a voice sample to one of five locations. Findings of research that explores human listeners’ accuracy when attempting to identify the provenance of vocal stimuli in forced-choice tasks points to wide variation in this ability, ranging from recognition rates above 90% (Bush, 1967, p. 25) to rates of 30% or less (Williams et al., 1999, p. 351; Clopper and Pisoni, 2006, p. 212). There seems to be little consensus on what constitutes a ‘good’ or ‘bad’ recognition level, and the variation in recognition rates poses potential problems for fields such as language attitudes, which appear to rely in listeners’ ability to first identify a speaker before drawing on stereotypes based on this recognition (cf. Dragojevic et al., 2018). Variation in recognition rates has been shown to be conditioned by three main factors: the familiarity with target accent(s), the regionality of target accent(s) and the specificity of listener placement.

Familiarity, taken here to mean experience or exposure to an accent, can be seen to be an important factor in voice perception from a young age. For example, Jusczyk (2000, p. 80) describes how infants of 6 months old can discriminate phonemic contrasts “not present in their ambient language” (Clopper and Pisoni, 2004, p. 31). While infants lose this ability as they attune to their native phonology, and although phonetic discrimination is not the same as voice recognition, this early phonetic sensitivity still points to the importance of fine-grained auditory discrimination in later life. Amongst adults, the role of familiarity in accent identification has been examined in many studies (e.g., Preston, 1996a; Kerswill and Williams, 2002a; Clopper and Pisoni, 2004; Ikeno and Hansen, 2006; Baker et al., 2009). For example Ikeno and Hansen (2006, p. 402) show an almost perfect linear relationship between the familiarity with an accent and the ability of listeners to identify it. Baker et al. (2009, pp. 63–64) discuss familiarity in terms of two factors: region of origin and the amount of experience that listeners have with different varieties of a language, something also noted by Montgomery (2012) under the headings of ‘proximity’ and ‘cultural prominence’, as well as Preston’s (1996b) concept of ‘publicity’. ‘Proximity’ suggests that listeners will be more likely to recognize accents associated with their region of origin. ‘Cultural prominence’ and ‘publicity’ effects will tend to result in an advantage in recognizing accents that listeners have more experience of (see Braber et al., 2022, p. 20) due to increased mass/social media exposure of certain varieties (Montgomery, 2012). Mobility could also play an important role, as it will result in a greater amount of experience with speakers of other varieties due to contact with them (for example the “army brats” in Clopper and Pisoni, 2004).

The ‘regionality’ of an accent, or its difference from the ‘standard’ is a further factor that affects the ability of human listeners to locate it (Hanani et al., 2013, p. 72). Of course, in English there is no spoken standard, although there is a general folk perception of the existence of one, and the term ‘Standard Southern British English (SSBE)’ is widely used (Mompeán, 2025). On this basis, it is reasonable to assume that there would be a cline of ‘locatability’ based on the difference from the SSBE. Difference in general (not only in relation to the standard) is also clearly an important factor in listeners’ ability to accurately discriminate between different accents. We return to the linguistic information present in a voice sample and the ways in which this is used in the following section.

Specificity is a further factor that can be used to understand human listeners’ accurate identification of accents. The fact that infants can distinguish between ‘home’ and ‘not home’ language, noted above, points towards a general ability of human listeners to make broad distinctions between the different accents they may encounter. Such broad distinctions have been tested in perceptual experiments focusing on L2 listeners’ perception (e.g., McKenzie et al., 2019). In L1 perception, specific recognition of variation within countries can be more difficult for human listeners. For example, although van Bezooijen and Gooskens’ (1999) research showed that recognition levels for UK language varieties were high based on broad regions (88% of listeners successfully allocating samples to these regions), accuracy fell to 52% when listeners were asked to allocate them to smaller areas (van Bezooijen and Gooskens, 1999, p. 40). A similar pattern was found in Braber et al. (2022), with listeners generally unable to place samples from locations in Scotland or Ireland with any more specificity than the country of origin.

Finally, there may be social factors that could interfere with the accurate placement of voice samples. Montgomery (2011), for example, shows that listeners may appear to misidentify samples that they recognize are similar to their own variety if they hold negative attitudes towards the speaker. This is similar to findings of ‘claiming’ and ‘denial’ effects found in Williams et al. (1999) in which favorably regarded voices were allocated to respondents’ home areas, and negatively rated voices appeared less likely to be. These findings suggest that accent recognition is not simply a matter of listeners’ matching linguistic features to a possible speaker location, which is what is suggested happens when listeners make judgements about speakers (see Dragojevic et al., 2018). Instead, there is a complex relationship between what is played to listeners, what they attend to, the meaning(s) they associate with the feature(s) attended to, and the likelihood of these meanings being in some way related to the geographical origin of the speaker.

### 1.2 Linguistic differentiation of accents in England

Perceptual research in England has used a rather broad categorization of what might be important for listeners as they attempt to locate speakers, largely based on production data that suggests regional distinctions characterized by variation in a small set of features. The most obvious of these are features that typically vary between Northern and Southern varieties of English in England. These are all vocalic features, and include the vowels in the bath, strut, face, and goat lexical sets (Wells, 1982a). It has long been observed that prominent North–South shibboleths in England are the vowels in bath and strut words (Wells, 1982b; Chambers and Trudgill, 1998; Wales, 2006; Vriesendorp, 2021). In the North the bath vowel is merged with the trap vowel, whereas there is no merger in most of the South [in the South West of England, the picture is different, as discussed by Moore and Carter (2015)]. A similar merger is evident for strut and foot in the North of England, where there is none in the South of the country. Both distinctions are stable (see Blaxter and Britain, 2021).

Newer North–South distinctions may be emerging too. It has been argued that varieties of Northern Englishes in England have increasingly exhibited dialect levelling, where speakers move from local forms to supralocal forms, sometimes called ‘General Northern English’ (Watt, 2002; Cardoso et al., 2019; Strycharczuk et al., 2020). For example, Watt (2002) showed that local centering diphthongs in the face and goat lexical sets were being avoided in Tyneside English, with speakers moving towards monophthongal forms more common in much of the rest of the North of England and classifying their own accents as ‘Northern’ rather than ‘Geordie’ (Tyneside English). Lawrence (2015a: 43) suggests that monophthongal face and goat vowels are characteristic of Northern English accents, contrasting with diphthongal forms in the South.

Lawrence (2015a) tested the geographical perception of bath, strut, face, and goat vowels using single-word stimuli and free-choice map placement tasks with 86 listeners. For each variable, variants typical of northern varieties led respondents to place speakers in more northern locations, while Southern variants cued more Southern placements (all *p* < 0.001). This suggests that these features are available for listeners to make general judgements about the locations of speakers, a finding corroborated by Vriesendorp (2021). Although Lawrence’s research suggests that many listeners find it relatively easy to locate speakers based on single words, it is worth noting that the task respondents were asked to undertake is somewhat removed from how listeners encounter speech in everyday life. In natural speech listeners will encounter a constant stream of phonetic information that they must process and ascribe meaning to. Other research has attempted to consider this more closely and has examined listener sensitivity to features embedded in longer stretches of talk. Levon and Fox (2014), for example, replicated Labov et al.’s (2011) study of the ‘sociolinguistic monitor’, but found that listeners were generally lacking in sensitivity to the frequency of -ing variation or th- fronting. This suggests a lack of awareness of certain features, or a hierarchy of salience for features. We return to salience below.

Further research that has attempted to examine perceptions of linguistic features in a more naturalistic setting was conducted by Watson and Clark (2013, 2015) using a slider-based audience response method. This approach sought social evaluations of speakers in real-time as they heard listeners. They were able to demonstrate coincidences between instances of the nurse-square merger and significant slider movements for Liverpool speakers and listeners, suggesting that this feature of the variety has some salience. More general research in this paradigm has suggested that there is little similarity between listener perception of specific features and evaluations (Levon et al., 2022).

Montgomery and Moore (2018) and Moore and Montgomery (2018) used an early version of the method used in the present research to examine listener attention to specific accent features as they heard a speaker from the Isles of Scilly recount two different accounts of life on the islands. They found that listeners attended to different features depending on the account that they were listening to. In particular, they demonstrated that features could become attended to depending on the broad context in which they are found (i.e., in the guise in which listeners encounter them). We will return to this idea in the sections below.

There is no doubt that English accents remain varied and that studies of production data demonstrate important differences. For listeners, however, the features that are important to them as they attempt to locate a speaker can be heavily influenced by the task type. More experimental and targeted research does point towards a general ability to place speakers based on very little stimulus data. Research that takes a less targeted approach, however, reveals that there is less consistency about the features that might be considered important by listeners, and therefore it is difficult to predict precisely which features might be used by them as they attempt to work out where a speaker is from. An important implication of this is that it is difficult for researchers to be confident about which features they might want to focus on when conducting research of this type. This is one of the justifications for the use of the approach we detail below.

### 1.3 Salience and attention

As many have noted (e.g., Kerswill and Williams, 2002b; Meyerhoff, 2011; Alderton, 2020),‘salience’ is a term that has wide currency in sociolinguistics, but which has historically been relatively underdefined. The term has been connected to others such as ‘noticeability’ and ‘awareness’ (Drager and Kirtley, 2016, p. 12). ‘Awareness’ is considered by Drager and Kirtley (2016, p. 1) to relate to “one’s consciousness of events or experiences,” and Nycz (2016, p. 64) defines ‘noticeability’ as the “conscious awareness and subjective experience of a linguistic feature.” The literature on perceptual dialectology also provides useful ways of conceptualizing awareness, particularly through Preston’s work on folk linguistic awareness (Preston, 1996b). In particular through Preston’s (1996b) account of folk linguistic awareness.

Salience is therefore linked to listeners’ conscious experience of linguistic features. Through the use of the term ‘salience’, then, sociolinguists have really been seeking to understand the extent to which a linguistic feature might be important, or “stick out” (Alderton, 2020: 74), for listeners in some way, as well the underlying reasons for this. In recent years, some consensus appears to have been reached in order to accommodate two different types of salience that relate to language-internal factors (‘cognitive salience’) and language-external factors (‘social salience’) (Kerswill and Williams, 2002b; Alderton, 2020, p. 74). Auer (2014) proposes a third type of salience. His ‘physiologically determined salience’ relates to instances in which words are produced loudly, for example, although in this paper we subsume this type of salience into the language-internal (cognitive) salience type.

Cognitive salience “refers to the property of a stimulus that makes it stand out relative to other stimuli” (Hogg and Vaughan, 2017, p. 63). This has a contextual element, as certain linguistic items will have cognitive salience due to their volume, pitch, or position (Drager and Kirtley, 2016, p. 12), and therefore may have salience in situations where a contrast occurs (i.e., when a particular sound is louder than those which surround it (cf. Auer, 2014)) but not in situations in which they do not. This means that a segment has cognitive salience if “it has a large surprisal value when compared to an array of language input” (Rácz, 2013, p. 51). This intrinsic type of salience was seen as analogous to ‘awareness’ by Drager and Kirtley (2016).

Socially salient linguistic features are those whose salience derives from their indexical links with certain social meanings. This means that some features exhibit social salience due to enduring properties, such as their regional distinctiveness or ‘localizedness’ (see Honeybone and Watson, 2013; cf. Hanani et al., 2013) in comparison to perceptions of a ‘prestige’ or ‘neutral’ accent. A ‘neutral’ accent is likely to be the perceiver’s own, as numerous perceptual dialectology and language attitudes studies have found (e.g., Montgomery, 2012; Sharma et al., 2022).

As noted above, Montgomery and Moore (2018) demonstrate a dynamic aspect to social salience. This means that some features may have social salience due to matters of ‘surprisal’ in relation to the stimulus in which they are found, due to a lack of congruence with expectations about what ‘should’ be present in a stimulus. For example, Montgomery and Moore (2018) show that the unexpectedness of rhoticity when a speaker was discussing island rituals versus when they were talking about farming topics meant that it was significantly more likely to be attended to in the former recording than the latter (given the strong indexical links between farming, rurality, and rhoticity in England). These findings open up the possibility that cognitive salience does not always have to be present for social salience to be observed (contra Auer, 2014; Juskan, 2018), as it was the social context that provided the frame in which salience could be demonstrated, and rhoticity was present in both of their test guises in Montgomery and Moore (2018).

The possibility that social salience can sometimes operate without cognitive salience (i.e., that a social meaning of a particular feature might be more important than the properties of its realization) means that we need to think more carefully about social salience. We aim to do this in the present paper. As part of this we argue that social salience is a gradable phenomenon. That salience is gradable is perhaps news to no one. The simple concepts of reading speed or loudness and their relationship to the extent to which words are spoken more slowly or loudly will be more cognitively salient makes this clear. This is also implicit in some early accounts of salience, for example Trudgill (1986, p. 38) who mentions the concept of “extra strong salience” and often includes adverbial modification of ‘salient’ (e.g., ‘too salient’, ‘highly salient’) over the course of his text.

Trudgill (1986), however, makes no explicit distinction between the different types of salience he might be referring to in this work, although phrases like “too salient” (Trudgill, 1986, p. 18) suggest that social salience better captures his thinking. Later, Juskan (2018, p. 43) evokes the idea of a “very high degree” of social salience when hypothesizing which variables might be capable of evoking priming effects. We build on these ideas in this work and will consider the degree of social salience as a factor when explaining and interpreting our results.

We do not claim to be measuring salience in this research, however. As we explain in the methodology section below, in this paper we explore listener attention to features as listeners try to work out where speakers might be from. We will then discuss the ways in which different types of salience contribute to this attention. Salience in this research, therefore, is taken to be explanatory of the measurable property of attention (following Montgomery and Moore, 2018).

Our focus on attention as a measurable correlate of salience also requires us to consider how non-linguists consciously describe what they hear. Preston’s (1996b) notions of availability and detail, which are two of his ‘modes of folk linguistic awareness’ (Preston, 1996b), are particularly relevant. His cline of availability captures the extent to which features are open to comment: some (such as certain phonological rules) remain entirely ‘unavailable’ to non-linguists, others emerge only through explicit prompting, while ‘common’ features form part of everyday discussion. The notion of detail reflects the specificity of listener comments, ranging from broad, global remarks (e.g., ‘the accent’) to precise references to features such as th- fronting or h-dropping. Together these modes suggest that listeners vary in both their ability to access particular features for accent recognition and their capacity to describe them in detail. Listeners may classify a speaker’s accent region without knowing (or being able to articulate) the basis for their judgement. Nonetheless, it remains important to examine which features are available to listeners and how explicitly they can characterize them.

## 2 Materials and methods

### 2.1 EDA corpus

The stimuli for this study were drawn from the English Dialects App (EDA) corpus (Leemann et al., 2018). The English Dialects App is based on technology used in the Dialäkt Äpp project in Switzerland (Leemann et al., 2016). Both applications attempt to crowdsource dialectological data via smartphones. They invite users to provide responses to questions as part of a ‘dialect quiz’ that aims to elicit specific phonetic, lexical, and morphological variants, with the objective (for users) of having the app ‘guess’ their location from their answers. This gamification increases engagement with the apps and thus a greater amount of data collection based on the responses provided by users. Data collected by apps of this type can be compared with pre-existing data from more traditional studies (e.g., Leemann et al., 2016; Britain et al., 2020).

The English Dialects App introduced further functionality in addition to the ‘dialect quiz’, adding the ability for users to add a recording of themselves reading the 10 sentences that make up the short story ‘The Boy who Cried Wolf’. The 10 sentences comprising this story can be found in the Supplementary materials. ‘The Boy who Cried Wolf’ is a passage that provides a good range of the sounds of English and permits straightforward measurement of monophthongs, and has fewer repeated words than other passages that could be used for descriptions of English pronunciation (Deterding, 2006, p. 193).

Strycharczuk et al. (2020) analyzed 105 speakers from five Northern English cities (Leeds, Liverpool, Manchester, Newcastle upon Tyne, and Sheffield). Using only vowel data, their random-forest models showed high classification accuracy for some speakers, identifying a set of ‘algorithmically salient’ cues that had not been widely studied in sociolinguistics. For the present study we take these same five cities and base our speaker selection on the prototypical voices that Strycharczuk et al.’s models had classified most reliably. This allows us to test whether the same cues that drove algorithmic accuracy are also those that human listeners notice in real-time accent recognition.

**Figure 1:**
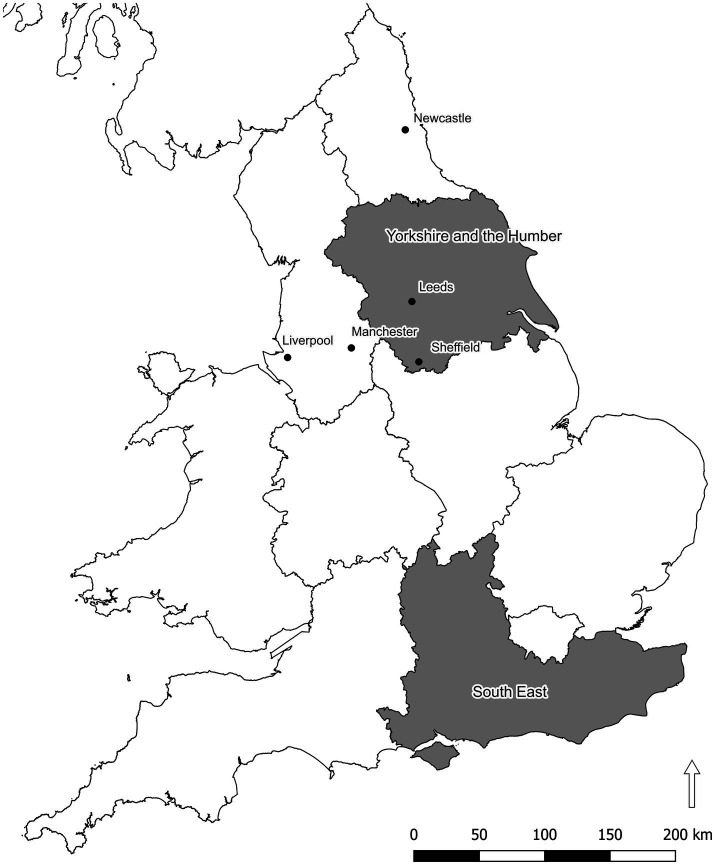
Location of five cities from which samples were selected. The two regions of England from which respondents were recruited are also marked. Contains National Statistics data © Crown copyright and database right 2018. Contains NRS data © Crown copyright and database right 2018. Source: NISRA: Website: www.nisra.gov.uk. Contains OS data © Crown copyright [and database right] (2018).

Figure 1 shows the location of the cities. Strycharczuk et al. (2020) used only those “recordings of sufficient quality, excluding those that were incomplete, had excessive background noise, multiple talkers present, etc.” (Strycharczuk et al., 2020: 5). We based our selection of speakers for each city on this subsample of the EDA, using recordings that had previously been used by Strycharczuk et al. (2020), as we explain in the next section.

### 2.2 Sample selection and manipulation

Our sample selection follows directly from Strycharczuk et al. (2020). We used the same five Northern English cities, and within each city we selected one of the speakers whose recordings their random-forest models had successfully classified. This ensured that our human listeners were exposed to voices that the algorithm considered prototypical for each city, allowing us to test whether the cues enabling machine classification would also underpin human recognition.

We selected one speaker per city to keep the experiment a manageable length and to avoid participant fatigue (including more speakers would have made the experiment overly long and would likely have reduced data quality). Out of the samples most accurately recognized by the random-forest models, we then chose recordings that were most homogeneous in terms of gender, ethnicity, age, and reading speed of the speakers, whilst avoiding reading styles which were (impressionistically) more marked. The selected samples were recognized by the random forest models as the correct variety in 100% of cases for the Manchester, Liverpool, and Newcastle samples, and in 97 and 87% of cases for the Leeds and Sheffield samples, respectively. This process means that we were able to be confident that the samples played to participants were representative of the locations they were from. All speakers were white women between the ages of 20 and 31. The audio samples were between 67 s and 79 s long.

Using Praat (Boersma and Weenick, 2025), we identified the start and end of each sentence using voice activity detection. We added 0.2 s of silence at the start and end of each sentence to ensure a consistent amount of silence between sentences and then concatenated the sentences into a single recording. A series of beeps (3 short, 1 long, at 1 s intervals) was added to the start of each recording to alert the listener that they were about to hear the passage. The samples used in the experiment can be made available on request.

### 2.3 Procedure

**Figure 2:**
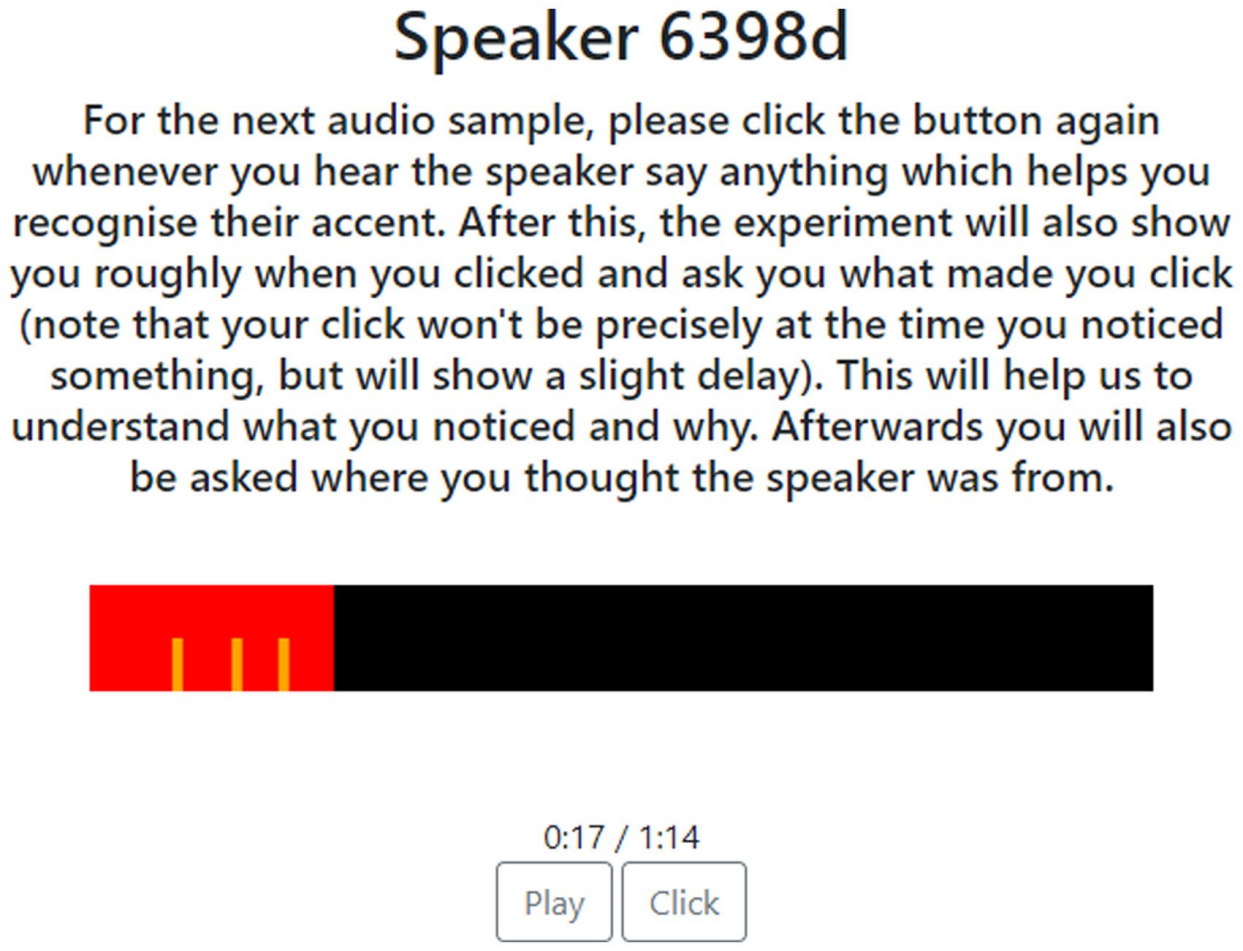
The SLIC real-time interface. Clicks that have been made are indicated by orange lines, and the red bar shows the progress of the audio sample.

**Figure 3:**
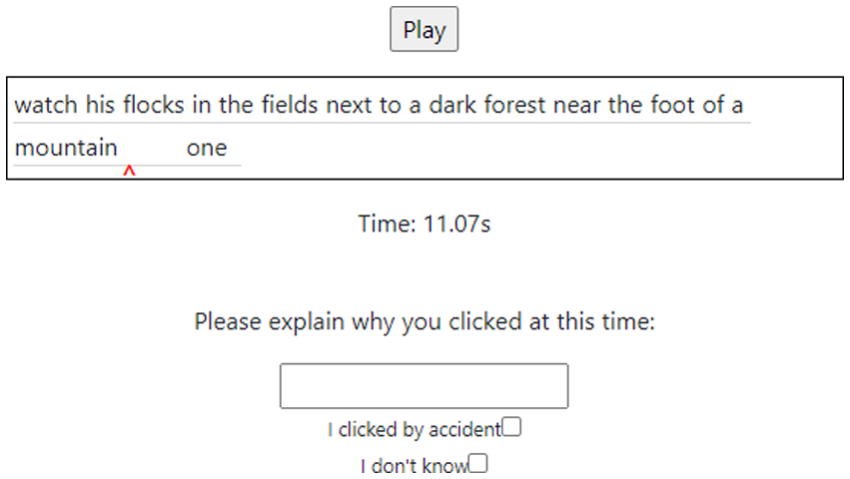
The SLIC review interface, showing the transcription fragment, the play button to play the accompanying audio fragment, a small red arrow indicating the location of the click, and the options for providing the justification for the click.

The research presented in this article used the Salient Language in Context (SLIC) web app (Montgomery et al., 2025) to collect data, deployed to participants recruited through Prolific. SLIC is a development of the approach first used by Montgomery and Moore (2018), that permits the collection of real-time attention to speech data, along with contextual data which helps to explain the attention data. Real-time attention data are gathered by asking respondents to use a mouse to click on a button each time they attend to a feature of interest as they hear a sample being played (See Figure 2). Once the sample has finished playing respondents are then invited to provide reasons for the mouse clicks they have made with the help of a transcript fragment and the accompanying audio fragment (Figure 3). The result is a series of time-aligned attention events paired with freely provided justifications for each of these events.

Participants were asked to complete the task on a desktop or laptop computer. It was suggested that they used headphones during the task, and that they completed it in a quiet room with no distractions. We first collected demographic data from respondents (age, gender, location, mobility and education), before they moved on to the real-time attention and review tasks proper. Listeners were first played a practice calibration sample where they listened to a speaker from London who exhibited th-fronting and were asked to click at any point in the sample when they heard examples of th-fronting. This provided listeners with a practice run with the interface, as well as priming them to listen for regional accent features. After this the participants were presented with the critical audio samples in randomized order.

For each sample, participants were provided with instructions, which remained on screen as they listened to the speaker. This interface, including the instructions, is given in Figure 2.

After hearing a sample and making their clicks, the screen refreshed (removing the click interface) and participants were shown a list of each time they clicked, along with a transcript of the excerpt in which they clicked as well as the playable sound file of the excerpt. For each click respondents were invited to type what they heard which made them click, or to indicate they made a mistake when clicking or do not know why they clicked. Figure 3 shows this element of the experiment. The final task for each speaker was to select which place they thought the speaker was from using a drop-down list of the five relevant cities. This placement option was only shown to respondents if they had added one or more clicks for the sample as they listened.

### 2.4 Respondents and demographics

We deployed two identical versions of the experiments, both aimed at recruiting monolingual English participants with no speech, hearing, or language difficulties. Using Prolific, we targeted one experiment at participants from the South-East of England and the other at participants from the Yorkshire and the Humber region. Figure 1 above shows the location of these participants. Hundred and twenty-two participants completed some aspects of the study, although not all completed it. We examined the completed dataset thoroughly, discarding incomplete attempts, or attempts at the task that provided no data. After this initial pass at the dataset, we had 98 complete responses that were usable for analysis. Participants were paid using Prolific at an average rate of £10.27 per hour. Participants spent a varying amount of time completing the experiment, from 10:58 to 66:32 (mean 23:06, standard deviation 11:00).

We aimed to collect an even number of responses from the two regions of interest. Our final dataset roughly achieved that aim, with 51 listeners from the South-East of England and 47 from Yorkshire and the Humber. The sample had a similar balance between men (*n* = 50) and women (*N* = 48). Participants had a mean age of 41.4 years old (SD = 13.4). We collected further background data from participants, including highest Education level and the number of house moves they had undergone in the past 10 years (as a proxy for mobility, although we do note that this may be a flawed measure as people can move both within and outside of the region of their birth).^1^ We do not use these demographic data to analyze the data in what follows, save for region.

### 2.5 Data coding and processing

Data were downloaded from the SLIC tool as Excel files containing tabs for overall demographics and user data, and then for each sample there was a tab for click and comment data, plus a further tab containing the placement data. In total there were 2,476 clicks and comments (either justifications, ‘do not know’, or ‘mistake’) made in response to the samples. Rows of data for clicks were provided for every click made for each sample. For each click the full text justification for the click as typed by the respondent was provided, or there was either an indication that the listener made a mistake (‘accident’) and had not meant to click where they did or that they were unable to tell why they had clicked when they did (‘dk’).

Two of the authors of this paper independently coded the data, with differences flagged and resolved. Comments that mentioned vowels were coded according to Wells (1982a) lexical sets, with comments related to consonants coded using the IPA consonant symbol as appropriate. Some comments were coded more than once. For example, a respondent to the Liverpool sample clicked at 51.53 s and gave the comment “loochin for,” which was coded as k and as -ing (given the respelling of ‘k’ and ‘ing’). We use other coding categories when comments did not relate to segmental features (‘intonation’, ‘lexical’), when comments were either not specific enough about what was being referred to or were irrelevant (‘unclear’), or when comments were not classifiable for other reasons (‘other’). Clicks that had the value ‘intonation’, ‘lexical’, ‘unclear’, ‘other’, ‘accident’, or ‘dk’ were excluded from the dataset we use in this article. Setting a high bar by only counting features that were clicked and then commented on in a specific enough fashion to code for a feature in this way means that we can demonstrate a level of certainty about specific feature attention in our dataset. After this process we were left with 555 total coded clicks. Table 1 shows the structure of the data we analyze in this paper.

**Table 1:** Data structure for click and comment data.

Sample	Time	uid	Coded	Comment	Region
Newcastle	6.583015	100	cure	poor sounds like pooh-er	SE
Sheffield	6.611519	100	choice	boy almost like bay	SE
Sheffield	6.715581	61	cure	Poor - oor sound	YH
Newcastle	6.8328	64	cure	poor pronounced pua	YH
Newcastle	6.852088	42	strut	once = woonce	SE
(…)					

‘Sample’ refers to the voice sample the click was made for, ‘time’ is the time of the click in seconds, ‘uid’ is the respondent number, ‘coded’ is the accent feature a participant comment was coded as, and ‘region’ gives the location of the listener.

Clicks which occurred within 5 s of the end of a previous token of that variable (bath, goat, t, etc.) were included in our dataset. The vast majority of clicks and coded comments occurred within this delay threshold (*n* = 516, 92.64%). Applying our high threshold for inclusion in the dataset which prioritized our ability to pinpoint the features that listeners were able to attend to and identify, plus an additional time-based threshold, meant that 20.84% of the clicks made by listeners while they listened to the accent samples were useable for the purposes of the analysis we wished to perform in this paper.

## 3 Results

### 3.1 How good are listeners at placing speakers from the five Northern English cities?

Listeners made over 90 placement attempts for each of the samples they heard. As noted above, when listeners did not provide click data, they were not invited to place the speaker. Some listeners provided click data for some samples and not others, resulting in 93 placement attempts for the Leeds, Liverpool, and Newcastle samples, and 92 for the Manchester and Sheffield speakers.

**Figure 4:**
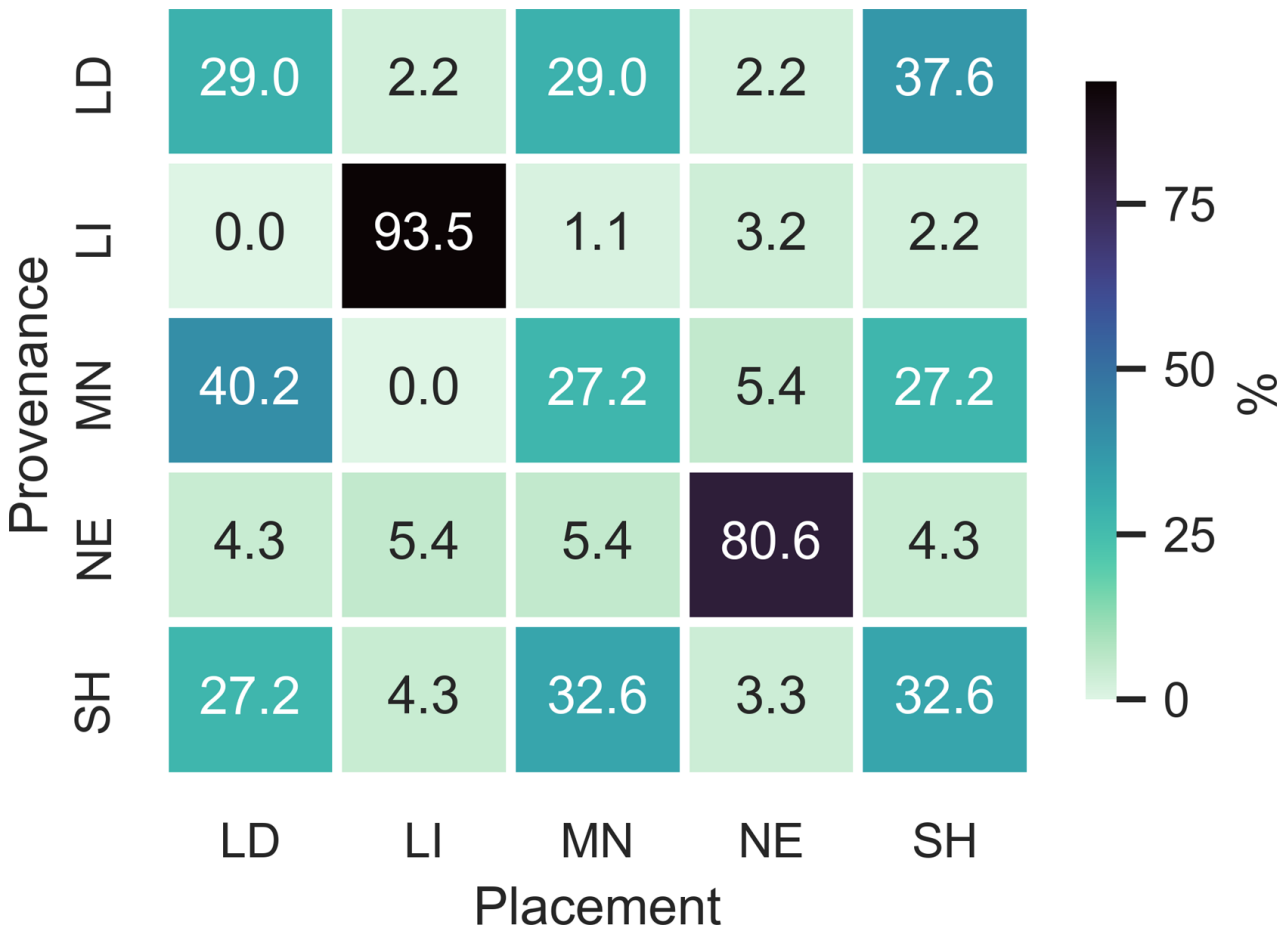
Matrix plot showing placement (*x*-axis) of samples (with provenance on the *y*-axis) for all respondents for Leeds (LD), Liverpool (LI), Manchester (MN), Newcastle-Upon-Tyne (NE), and Sheffield (SH).

Figure 4 shows a visual representation of the placement attempts for each of the voice samples for all listeners. It reveals that respondents were relatively good at placing the speakers from Liverpool and Newcastle upon Tyne. For Liverpool, the overall correct placement percentage was 93.5% and for Newcastle it was 80.4%. It is clear, therefore, that the placements for these speakers were largely accurate, and well above chance (i.e., 20%).

Conversely, listeners placed the other three speakers less accurately and it is of note just how poor our listeners were at the placement task for the Leeds, Manchester and Sheffield samples. Most listeners were able to correctly identify speakers from Liverpool and Newcastle. This means that when listeners who could correctly place the Liverpool or Newcastle samples heard a speaker that they could rule out as representing one of these locations, they had three other possible choices. Accordingly, each of these three samples had around a 33% chance of being selected by chance by those listeners.

**Figure 5:**
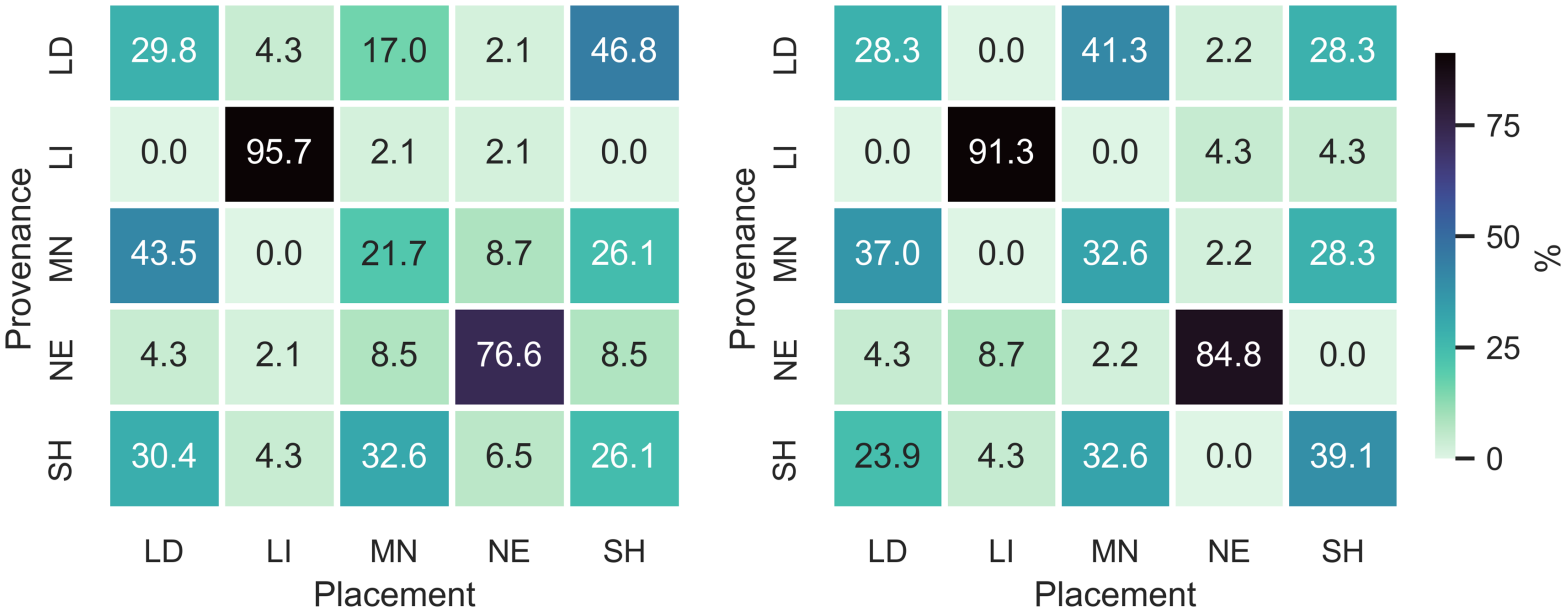
Matrix plots showing placement (*x*-axis) of samples (with provenance on the *y*-axis) for respondents from the South East of England (left) and Yorkshire and the Humber (right).

We tested the effect of any demographic effect on listeners’ recognition skills for the 5 accents by means of mixed effects logistic regression modelling. This included the dependent variable of whether a participant’s response was correct as well as the fixed effects of the guise in interaction with the region of the listener, and two mobility variables (travel to work and moving history). Education, age and gender were included as fixed effects (without an interaction) and participant ID as a random effect. Using the package ‘buildmer’ (Voeten, 2025) a best-fit model was selected where all terms were significant. The best fit model only included the guise but no demographic factors and no random effects. When participant region was added to the best-fit model as a fixed effect it did not improve the model significantly (z = 1.076, *p* = 0.28). Figure 5 demonstrates this visually.

We now turn to the question of the features that listeners attend to as they make decisions about where they think a speaker is from.

### 3.2 Which variables are most attended to by listeners?

As a preliminary to presenting that data, several aspects of the question need to be clarified. First, we will consider variables, in the first instance, without taking into account the context in which those variables occur (for example, the position at which the segment occurs in a syllable, word or intonation phrase). Examples of the variables we will go on to discuss are the strut vowel, /t/, −ing, etc. Second, when we refer to variables being attended to, this means the occurrence of a click which was then commented on by the listener, and that comment was subsequently coded as relating to a particular variable or to particular variables, as explained above in relation to coding and processing. Third, when we refer to variables which are most attended to, we will consider several different measures to be dealt with in the below. Table 2 shows the total number of clicks and coded comments for each variable, across all samples. Note that both Tables 2, 4 that we introduce later do not consider the fact that the variables occur with different frequencies, although we return to this later in the paper (Table 3).

**Table 2:** Number and proportion of clicks and coded comments received for each variable, all samples combined.

Rank	Variable	*n*	%
1	strut	75	14.53
2	k	61	11.82
3	t	51	9.88
4	ing	32	6.2
5	bath	29	5.62
6	happy	26	5.04
7	h	25	4.84
8	face	23	4.46
9=	goat	21	4.07
9=	foot	21	4.07
11	GOOSE	15	2.91
12	mouth	13	2.52
13	cure	12	2.33
14=	s	11	2.13
14=	ng	11	2.13
14=	lot	11	2.13
14=	r	11	2.13
18=	letter	8	1.55
18=	nurse	8	1.55
18=	price	8	1.55
21	school	7	1.36
22	th	6	1.16
23=	fleece	5	0.97
23=	north	5	0.97
25=	trap	4	0.78
25=	schwa	4	0.78
27	n	3	0.58
28=	tr	2	0.39
28=	square	2	0.39
28=	start	2	0.39
31=	f	1	0.19
31=	choice	1	0.19
31=	ch	1	0.19
31=	thought	1	0.19
	Total	516	

**Table 3:** Results of a two-sample Kolmogorov–Smirnov test to determine if there are differences in the distribution of listener reactions by the SE and YH groups.

Sample	Test statistic	*p*-value
Leeds	0.080	0.197
Liverpool	0.032	0.991
Manchester	0.130	0.003**
Newcastle	0.038	0.940
Sheffield	0.047	0.844

**Table 4:** Number of clicks and coded comments received for each variable for each sample, and proportion of all clicks and coded comments received for each variable for each sample (S-PROP).

Variable	Leeds		Liverpool		Manchester		Newcastle		Sheffield	
	*n*	S-PROP (%)	*n*	S-PROP (%)	*n*	S-PROP (%)	*n*	S-PROP (%)	*n*	S-PROP (%)
bath	8	15.38	5	2.99	6	8.33	4	3.36	6	5.66
ch			1	0.6						
choice									1	0.94
cure	2	3.85					9	7.56	1	0.94
f			1	0.6						
face			3	1.8	1	1.39	5	4.2	14	13.21
fleece			2	1.2	1	1.39	2	1.68		
foot	3	5.77	2	1.2	1	1.39	9	7.56	6	5.66
goat	4	7.69	1	0.6	3	4.17	1	0.84	12	11.32
goose	1	1.92	2	1.2			9	7.56	3	2.83
h			8	4.79	6	8.33	5	4.2	6	5.66
happy	2	3.85	2	1.2	6	8.33			16	15.09
ing			12	7.19	2	2.78	17	14.29	1	0.94
k	2	3.85	53	31.74	1	1.39	4	3.36	1	0.94
letter			1	0.6	1	1.39	2	1.68	4	3.77
lot	3	5.77	3	1.8			2	1.68	3	2.83
mouth	1	1.92	6	3.59	1	1.39	5	4.2		
n			2	1.2			1	0.84		
ng			1	0.6	6	8.33			4	3.77
north	2	3.85	7	4.19			3	2.52		
nurse			2	1.2			1	0.84		
price	2	3.85					1	0.84	3	2.83
r			9	5.39	2	2.78				
s			10	5.99	1	1.39				
school			1	0.6	4	5.56	1	0.84	1	0.94
schwa	1	1.92					3	2.52		
square			1	0.6					1	0.94
start			12	7.19	1	1.39			1	0.94
strut	17	32.69			19	26.39	8	6.72	19	17.92
t	3	5.77	11	6.59	9	12.5	26	21.85	2	1.89
th			6	3.59						
thought							1	0.84		
tr			2	1.2						
trap	1	1.92	1	0.6	1	1.39			1	0.94
Total	52		167		72		119		106	

Table 4 shows the number of clicks and coded comments for each variable, arranged by sample. The table also shows the proportion of the clicks and coded comments for that sample relating to that variable (we will refer to this measure as S-PROP).

**Figure 6:**
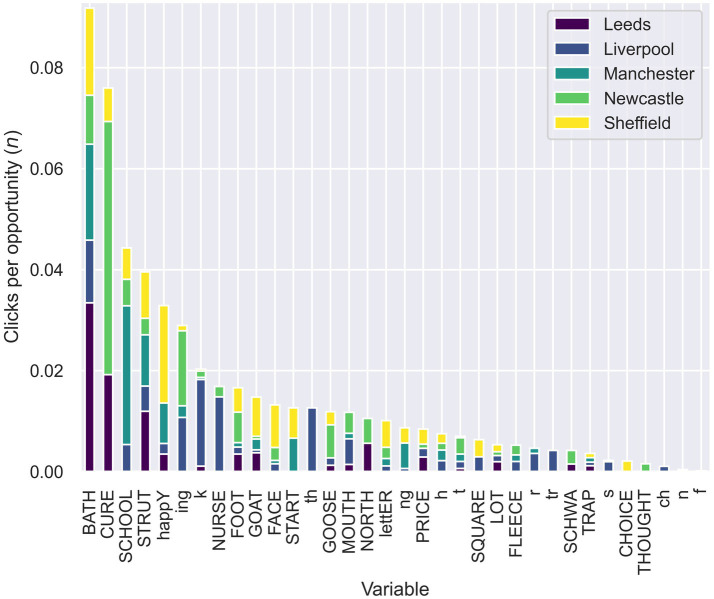
Number of clicks and coded comments received for each variable coming from each sample (V-PROP).

Figure 6 shows the proportion of all clicks and coded comments for each variable coming from each sample (we will refer to this measure as V-PROP). The figure deals with ‘clicks per opportunity’, which is the total number of clicks that were made by respondents of the total possible number of clicks. In Figure 6, total bar height reflects clicks per opportunity for that variable in all samples combined and the colored portions within each bar reflect the proportion of those clicks coming from each sample. The variables are ordered by number of clicks per opportunity for all samples combined. Clicks per opportunity is based on the number of responses containing at least one click and coded comment.

Our data collection method allows us not only to identify which variables get a click and comment, but also where in the time-course of the sample those click and coded comment responses occur. We are therefore able to identify ‘peaks’ in the raters’ responses (i.e., moments of particular activity). To identify peaks, we first identified how many clicks and coded comments there were within a 1 s window. That window was then moved through the utterance in steps of 0.2 s. The criteria for a peak are that the value of the current frame must be higher than the value of (i) the previous frame with a different value, and (ii) the next frame with a different value to the current frame. We are concerned here only with peaks of four or more clicks and coded comments. Table 5 shows the number of times a variable gets a peak across all samples.

**Table 5:** Number of peaks of four or more clicks and coded comments within a 1 s window, all samples.

Variable	*n*
STRUT	8
k	5
ing	4
t	3
ng	2
happY	2
BATH	2
r	1
NURSE	1
MOUTH	1
h	1
GOOSE	1
GOAT	1
FOOT	1
FACE	1
CURE	1
Total	35

Table 6 shows the peaks of four or more clicks and coded comments, for each sample. The highest peaks are at the top of the table; where there are multiple peaks of the same height in a sample, the variables are listed in alphabetical order.

**Table 6:** Peaks of four or more clicks and coded comments within a 1 s window, arranged by sample; column t identifies the time of the frame in which the peak occurs; column n identifies how many clicks + coded comments occurred in that peak.

Leeds			Liverpool			Manchester			Newcastle			Sheffield		
Variable	*t*	*n*	Variable	*t*	*n*	Variable	*t*	*n*	Variable	*t*	*n*	Variable	*t*	*n*
BATH	43.1	6	k	52.9	17	STRUT	18.3	7	t	12.9	9	happY	58.7	10
STRUT	39.3	4	k	42.4	6	STRUT	50.2	6	CURE	6.8	8	GOAT	26	9
STRUT	48.3	4	STRUT	20.8	6	BATH	44	4	GOOSE	15.6	7	STRUT	46	7
			k	8.3	5	ing	44.1	4	FOOT	12.7	4	happY	28.1	6
			NURSE	28.9	5				ing	50.3	4	FACE	63.5	4
			h	18.6	4				ing	63.5	4	ing	40.8	4
			ing	25.6	4				STRUT	20.6	4			
			ing	50.4	4				STRUT	53.2	4			
			k	10.6	4				t	20.5	4			
			k	50.3	4									
			MOUTH	25.1	4									
			r	11.2	4									
			t	11.9	4									

Table 2 reveals that the most attended to features were strut, k, t, ing, bath, happy, h, face, goat, and foot. The subsequent tables reveal, unsurprisingly, that these are not features attended to for all the five samples. Instead, some different features are attended to for the different samples. This is not always the case, however, and we present further results that permit us to expand our understanding of the data below. We will first consider the features attended to across all samples, before discussing features that were more likely to be attended to for specific samples, using a combination of the data in Tables 4–6 to select the features of interest.

### 3.3 Features attended to for all samples

Listeners attended to bath, strut, and goat across all samples. Bath and strut were notable as they were relatively close together in terms of the proportion of all clicks and coded comments for each variable coming from each sample (see Table 5). This signals the seeming importance of these features for each of the samples. In the discussion that follows, we examine the features that listeners attended to most readily and provide an account of the variable instances, characterizing them in terms of analysis conducted in Praat (Boersma and Weenick, 2025) for vowels (examining F1 and F2 values), and for consonants, coding performed independently by the paper authors. We also discuss matters such as the linguistic environment, the word in which features occur, and other pertinent details.

There were 2 examples of bath vowels in the reading passage, both occurring word-initially (in ‘afternoon’ and ‘after’). For all samples the two examples of the vowels were front, open, and unrounded. This realization is associated with the North of England (Wells, 1982b; Lawrence, 2015b). There was slight variation in the realization of the vowels in the Manchester and Newcastle samples, whereas the other samples’ realizations were extremely similar. The two instances of bath occur a good length of time apart from each other, at around 10 s and around 42 s into the sample. Ten seconds is very early into the sample, as 6 s was given over to the introductory ‘beeps’. This could explain the smaller number of clicks for the first instance of the feature (10 across all samples) compared to the greater number for the second (20 commented clicks).

There were 12 instances of strut vowels across the samples and they were typified by their back, close, and rounded quality, and demonstrated less variability across instances than other features (for example, happY for the Sheffield speaker). Accordingly, the strut vowels could all be described as typically ‘Northern’, per Wells (1982b). Instances of strut were not always attended to, despite the similar realizations noted above. Monosyllabic tokens were more typical (9 out of 12 instances) and were more likely to be attended to. The greatest attention was noted for the fourth and ninth instances of strut, in the words ‘fun’ and ‘duck’ (20 and 21 coded clicks respectively). The fourth instance of strut occurred around 2.5 s after the first three instances of strut, all of which occurred within 1.5 s of each other.

goat monophthongization is another feature (as with the previously mentioned variables) that is associated with Northern English accents (Lawrence, 2015a). Across the samples used in this experiment there are a range of realizations of this vowel from more to less monophthongal depending on the token number. There were 9 instances of goat in the reading passage, with the second example in the word ‘homes’ much more likely to be attended to than any other instance (this instance gained 16 of 23 overall clicks, and the next most clicked example, in the word ‘told’, gained three clicks). It should be noted that this second instance of goat is the most monophthongal for all of the speakers, although it has the shortest glide of all in the Sheffield sample. It is the Sheffield sample that contributes the greatest number of clicks for this variable overall (12 of 23 clicks).

A backed short bath vowel, a strut vowel that is similar in quality to the foot lexical set, and goat monophthongization are all strongly associated with Northern English pronunciation, as we discussed above. Given the samples played to respondents in this study, then, it is unsurprising that these features were attended to for all the samples (with roughly even V-PROP scores for bath and strut). This high attention for these features suggests that they have high social salience, indexing Northern English accents. It could be tempting therefore to assume that feature attention is inextricably linked to specific listener placements, and therefore that high feature attention can automatically be assumed to indicate high place-based indexical meanings. As we will explore below, however, this is not always the case. We start, however, with the Liverpool sample, where it seems that this assumption does hold.

### 3.4 Liverpool feature attention

Listeners recognized the Liverpool sample most accurately (see Figure 5). As well as demonstrating attention to the common features of bath, strut, and goat, there was also notable attention paid to specific features for this sample. The S-PROP calculation in Table 4 shows that 69.46% of the commented clicks for the Liverpool sample were for consonants (in other words, most of the commented clicks were for consonants for the Liverpool sample), compared to 44.54% for Newcastle, 37.5% for Manchester, 13.21% for Sheffield, and 9.61% for Leeds. We focus here on /k/, (ing), /t/, and /s/ as these are the features that gained more than 10 clicks and codable comments for this sample (see Table 4). We also consider nurse, as this was the vocalic feature with the highest V-PROP score for the Liverpool speaker (see Table 5).

**Figure 7:**
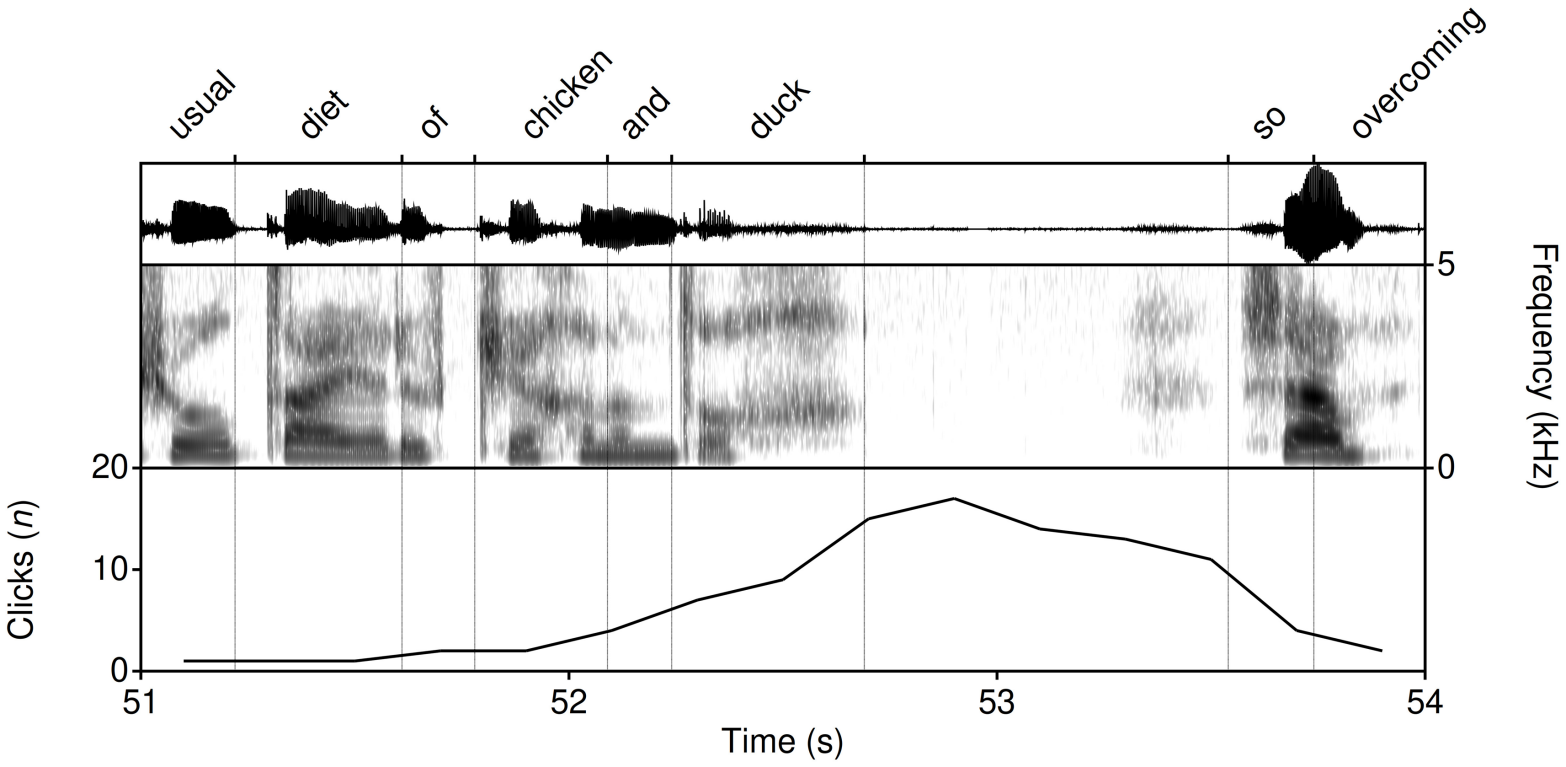
Listener reactions coded as responding to /k/ for the Liverpool speaker between 51 and 54 s.

There were 19 instances of /k/ in the reading passage. Listeners produced 86.9% of /k/ responses for the Liverpool sample (with the next highest being the Newcastle sample, which contributed 6.56% of the attention data for /k/). Of the total clicks for the Liverpool sample, 31.74% are for /k/ (*n* = 53). Fricated realizations of /k/ are widely understood to be particularly associated with Liverpool speech (see Watson, 2007a; Clark and Watson, 2016), and we coded 10 of the instances of /k/ as fricatives or affricated plosives. Of those features listed in Table 6 (showing click peaks of >4 clicks within a one second window), we coded all but one (at 8.3 s, in the word ‘flocks’) as a fricative or affricated plosive. The largest peak for /k/ came at 52.9 s, in response to the words ‘chicken’ and ‘duck’ in quick succession. Figure 7 shows this peak alongside the spectrogram and transcript between 51 and 54 s. These results demonstrate high attention for fricated and affricated realizations of /k/ in this sample.

Attention to (ing) formed a relatively high proportion of clicks for the Liverpool speaker, with an S-PROP value of 7.19%. It does not appear that the realization of the consonant is what affected listeners’ attention, however. We coded all consonants as [n]. They therefore do not differ from each other. Instead, the vowel preceding the consonant appears to have been more important in predicting listener attention. We coded two possible variant realizations of the vowel: [ə] and [ɪ]. We coded four vowels as [ə] and three as [ɪ]. It was only those examples of (ing) that contained a [ə] realization that garnered listener attention (in the words ‘shouting’ and ‘looking’).

We now turn to other features with high S-PROP values for the Liverpool sample: /t/, /s/, and nurse. In Liverpool, /t/, like /k/, is subject to lenition (Watson, 2007b). Honeybone (2001, p. 227) outlines a lenition trajectory in which the ultimate end point for the realization of voiceless stops is zero. Three of the 46 instances of /t/ in the Liverpool sample are zero realizations, and in the word ‘mountain’ we see a glottal realization. This is the instance of /t/ that is most attended to for the respondents (*n* = 6). Attention to /s/ is high for the listeners to the Liverpool sample, although it is unclear why this is the case. It is possible that fricatives are simply more prominent for this speaker due to the other significant (af)frication present in the sample. The final feature we discuss in relation to the Liverpool speaker is the nurse vowel. The speaker merged nurse with square in this sample, in a pattern typical of Liverpool speech (Watson and Clark, 2013). There are three instances of nurse, with the first example (in the word ‘heard’) gaining the greatest number of clicks and comments (five out of the seven clicks for this sample). The following examples of nurse have only one click each.

The importance of consonantal variation appears to be clear for the Liverpool speaker. The largest amount of attention across all samples was registered in relation to /k/, and other consonantal variables also appeared to be important for listeners. We now move on to the Newcastle sample, which was the second most readily identified, and which also demonstrated some significant patterns with respect to the attention paid to consonants.

### 3.5 Newcastle feature attention

The V-PROP value for /t/ was 50.98% for the Newcastle sample. Listeners most frequently attended to /t/ (S-PROP) for the Newcastle sample overall with 21.85% of coded clicks (*n* = 26). As noted above, there were 46 instances of /t/ over the course of the sample. The largest peaks of more than four clicks were for instance of /t/ at 12.9 s (9 clicks) and 20.5 s (5 clicks) into the Newcastle sample. These click peaks coincide with /t/ realizations accompanied by glottal reinforcement, a feature strongly associated with a Newcastle accent (Mearns, 2015). Other, smaller, peaks were also found when /t/ realizations were glottalized or glottal reinforced.

The cure lexical set can be diphthongal in Newcastle speech (Watt and Milroy, 1999, p. 27), and the Newcastle sample demonstrated a diphthongal realization of cure in the single example of this vowel (in the word ‘poor’, early in the reading passage). This token differed from the monophthongal or near-monophthongal realizations of cure in the other samples and explains why 75% of all coded clicks were for cure in the Newcastle sample. Goose was attended to more readily for the Newcastle sample than in the other samples (V-PROP = 60%). All clicks for the Newcastle goose vowel were in respect of the third instance of the variable, in the word ‘afternoon’. This instance of the vowel has a slight diphthongal quality for the Newcastle speaker, although it is notable that this is no more pronounced than other instances of goose in the sample (some of which were more diphthongal). It is the only instance of the vowel in a polysyllabic word, however. (ing) had a V-PROP value of 53.13% for the Newcastle speaker, with the highest peaks for instances three and seven (in the words ‘looking’ and ‘trying’, respectively). Example three of (ing) for the sample included a schwa vowel, and example seven demonstrated [ɪ]. Both consonants were [n], in line with recent research that has demonstrated that younger speakers in Newcastle prefer this variant (Mechler et al., 2022, p. 108).

We now turn to the Manchester and Sheffield speakers, where we describe patterns of feature attention despite a lack of accurate recognition for each sample. Such patterns suggest a cognitive salience explanation for the attention observed, but we will return to this in the section that follows.

### 3.6 Feature attention for Manchester, Sheffield, and Leeds

The school lexical subset was used in this research in order to capture the systematic variation present in _l environments in the wider GOOSE lexical set for speakers from Manchester. This results in diphthongal realizations for school in this area (Drummond et al., 2022). There was one instance of school in the reading passage (in the word ‘fool’ towards the end of the sample). This received commented clicks from four listeners for the Manchester speaker, which was more than for other samples (one click each for all samples except Leeds).

The Sheffield sample saw the highest levels of attention to the monophthongal realization of the goat vowel, as discussed above, as well as the happy vowel. Happy, in much of the North of England, is commonly realized as a lax vowel, with the notable exceptions of Liverpool, the East of Yorkshire, and the North East of England (Beal, 2010, p. 18). The lax happy vowel could therefore have been likely to occur in the Manchester, Leeds, and Sheffield samples. The Leeds sample showed no instances of laxing, with Manchester showing one and Sheffield demonstrating two. Happy in the second potential location (in the word ‘safety’) was lowered and centralized in both samples and had a lax quality in the fifth and final potential location for the Sheffield sample (in the word ‘unfortunately’).

The Leeds sample received little in the way of attention for specific features other than those mentioned above in relation to features noted for all the samples. This sample therefore differed to the samples representing Sheffield and Manchester. Having discussed the patterns in the data, and the features attended to overall, and by sample, we now turn to the importance of these findings in relation to salience, attention, and sociolinguistic perception.

## 4 Discussion

The results show a sharp contrast between the relative ease with which listeners identified the Liverpool and Newcastle speakers, and the much poorer performance for Leeds, Manchester, and Sheffield, where placement was at chance level. Importantly, this variation in accuracy was not explained by listener region, suggesting that the determining factor lay in the linguistic cues available rather than in who the listeners were. The feature-level analyses reinforce this point: certain well-documented Northern variables (e.g., strut, bath, goat) attracted attention across all samples, while distinctive consonantal and vocalic patterns (such as Liverpool /k/ lenition or Newcastle cure) aligned closely with the more successful placements. At the same time, the Sheffield happy vowels, while highly attended to, did not yield accurate recognition, underlining that salience alone does not guarantee correct placement.

Taken together, these findings highlight the complexity of the relationship between cognitive salience, social salience, and indexicality in accent perception. The Discussion now considers these patterns in more depth, examining what they reveal about listener attention, regional recognition, and the models of salience that can account for the data.

### 4.1 Attention and salience

The data we have presented in this paper confirm Montgomery and Moore’s (2018) finding that it is possible to measure feature attention in real time using naturalistic voice samples with the SLIC interface. Listener attention is structured according to the presence of specific features and is not random. Participants used the interface as intended to supply usable click and comment data, although depending on the sample this usable data could be as low as 20%. This points to the need to collect data from large numbers of respondents, as well as the potential impact of conducting the study online and therefore not under conditions in which listener attention could be more guaranteed. This is in line with the predictions made by Strycharczuk et al. (2020), which found similar difficulties in differentiating Leeds, Manchester, and Sheffield speakers using vocalic data. However, the poor location success amongst our listeners is perhaps surprising, as they had a greater number of linguistic levels available to them than the machine learning algorithm in Strycharczuk et al. (2020), which relied only on vowels.

#### 4.1.1 Placement patterns and regional effects

Turning to the patterns that we have uncovered, it is striking that there was very little difference in the responses from listeners in different locations in England, either in relation to the placement of voice samples or in the features that they attended to (see Table 3). This was particularly notable given the listener locations (the South-East of England and Yorkshire and the Humber) and the fact that they were hearing only speakers from the North of England. In relation to placement, this challenges Montgomery’s (2012) suggestion that proximity effects will result in a greater ability to recognize nearby speakers. One reason for this discrepancy may lie in task design: whereas Montgomery (2012) relied on free classification and map-labelling, the SLIC paradigm used here directs attention to specific instances within continuous speech, potentially reducing the influence of prior regional knowledge. More broadly, it may also be the case that since 2012 mobility and increased exposure to regional varieties through broadcast and social media have diluted the role of geographical proximity in shaping accent recognition. It is also worth noting that listener region itself played only a very small role in the features that were attended to, as it did in their ability to locate samples. The only case where responses diverged significantly between listeners from Yorkshire and the South-East was for the Manchester sample.

#### 4.1.2 Feature attention and the north–south divide

For feature attention, these results are also notable due to the importance of the North–South divide in England, and the indexing of this division by well-known linguistic shibboleths (i.e., bath and the lack of strut-foot split). One possible reason is that the exclusive focus on Northern voices in the present study meant that distinctive features were equally unfamiliar (or equally recognizable) to both listener groups. More broadly, given the prominence of the ‘divide’ in England, the fact that some features seem to have a universal social salience for listeners, indexing the linguistic north of the country, is perhaps less surprising than it might initially appear. Indeed, it is this ‘Northern’ meaning that is important. Wales (2006) documents the historical and contemporary othering of the North of England, and notes that linguistic features such as bath and strut stand “for the whole image” (Wales, 2006, p. 29) of the region. This almost synecdoche-like status of these features means that both Northerners and Southerners are likely to attend to bath and strut in particular when attempting to place the speakers in this experiment. Although we did not ask respondents to indicate if they did not consider samples to be other than Northern, post-test free comments did not mention that listeners were unhappy with the five city options they were presented with, meaning we can be confident that listeners knew that they were listening to voices from the North of England.

#### 4.1.3 Liverpool, Newcastle, and less recognizable samples

As we note above, in the cases of Liverpool (in particular) and Newcastle (for most respondents) the high level of correct recognition alongside the high attention rates for specific features in those samples means that we are able to understand for the first time the ways in which listeners use features to identify speakers as they hear them. We do not claim that single features work as shibboleths, but rather that a combination of features that listeners attend to is likely to result in an accurate placement. We also show the importance of consonantal features alongside vocalic segments in the correct identification of speakers. The Liverpool speaker, for example, saw high levels of attention for /k/, strut, (ing), /t/, and nurse and a very high placement accuracy. It must be hypothesized that these feature patterns contributed directly to the ability of most listeners to place the Liverpool sample correctly. Notable here is the marked focus on consonantal variables, which sets Liverpool apart from the other samples. Features identified readily in the Newcastle sample included /t/, (ing), cure, bath, and goose. 80.4% of listeners placed this sample correctly. The features that drew attention in the Newcastle guise are precisely those that have been well-documented in descriptive work on the variety, which no doubt contributed to the relatively high level of correct placement. We can contrast this with the samples for which there was less attention paid to multiple variants, for example the Sheffield sample which only saw high levels of attention for features other than bath, strut and face, for happy and goat (which itself was attended to for all other samples to lesser extent). The Sheffield speaker was placed with an accuracy level of no greater than chance by the listeners overall, as were the Leeds and Manchester samples. These samples had similar feature attention patterns, for different features, to the Sheffield sample. For instance, the Manchester speaker’s diphthongal school vowel was the only such realization across all five samples, which appears to explain the attention it received despite not supporting accurate recognition.

#### 4.1.4 Limits of feature attention

These findings tell us about the ways in which listeners use variation in the linguistic signal that they encounter to make place-based indexical sense of what they are hearing, but they also point to the limitations of listeners’ abilities at the same time. For Sheffield, for instance, the greatest attention was to the lowered and centralized lax happy vowels, particularly in *safety* and *unfortunately*. This aligns with Beal’s (2010, p. 18) observation that happy tensing is a salient marker in Northern varieties, differentiating Liverpool from Manchester and nearby towns. Yet in this case it did not yield accurate placement. Our data therefore show that listeners need to be able to triangulate what they are hearing to make accurate predictions about the location of a speaker. The data show that listeners will still attend to specific features that they consider to be salient in some way, but if there is less other confirmatory evidence to support specific social meanings they will be unable to interpret these features in a targeted socially salient fashion. This means that attention to individual features occurs independently of whether listeners recognize the sample. We noted in the Introduction that the cognitive salience for specific features is very often to do with the unexpectedness of the feature realization. Over the course of the discussion, we demonstrated that it is those features that differ from others in their lexical set (e.g., the lax happy vowels for the Sheffield speaker) that are more likely to be attended to. This is an important finding, both in relation to the ways in which listeners make place-based sense of what they hear but also in relation to language attitudes. There are two possible understandings of the language attitudes implications of this.

The first is an ‘error model’, drawing on Niedzielski and Preston’s (2009, p. 371) folk theory of language. In their framework, non-linguists often conceptualize language in terms of a hierarchy of correctness, with ‘The Language’ (i.e., a perceived standard) at the top. Speech encountered outside this idealized norm may be interpreted either as a recognizable dialect, or, if listeners cannot reliably link it to a known variety, as ‘errors’. We do not mean to suggest that listeners in this study literally labelled speech as erroneous; rather, the model highlights how forms that cannot be anchored in experience of a specific place may be understood negatively, as deviations from the standard. In this sense, what counts as an ‘error’ is unlikely to be uniform across listeners but will vary with the extent of their prior exposure to different accents. Experience is therefore central, as being able to categorize a speaker geographically requires not only hearing linguistic difference but also having sufficient familiarity with the social and regional associations of that difference. Without such knowledge, listeners may still attend to features, but their interpretations risk defaulting to more generalized or negative categories, which could in turn contribute to dialect discrimination.

A second possible model is that social salience is hierarchical, and that it also that it operates on a specificity cline. This would suggest that listeners will use more general dialect images to make judgments in cases in which social salience is less specific (i.e., for Sheffield, Leeds, and Manchester speakers), and more specific dialect images in circumstances in which social salience is more specific (e.g., for Liverpool and Newcastle speakers).

Our data demonstrates a hierarchy of social salience and allows us to understand social salience as a gradable phenomenon analogous to cognitive salience (for example the difference between something being loud versus very loud). We can see this most clearly in respect to the Liverpool sample, where the /k/ realizations are clearly highly socially salient (i.e., particularly socially indexical) whereas others (e.g., nurse-square merging) appear to be less so. Listeners appear to use these differing levels of social salience to help confirm that the speaker is indeed from Liverpool. We can see a different picture for the less recognized speakers. For example, the features highly attended to for the Sheffield speaker (correctly identified by only 31.5% of listeners) were strut (merged with foot), happy laxing, and face monophthongization. All these features are present in the Sheffield accent, but the same is true for several other traditional Northern English accents meaning that they have less diagnostic power for the listener trying to locate the speaker. In other words, they have less social salience.

#### 4.1.5 Alignment with sociolinguistic research

The findings of this research reveal that listeners attend most readily to features that are well-studied for each of the varieties examined in the experiment. Strut and bath have a long history of investigation as Northern English stereotypes (e.g., Chambers and Trudgill, 1998; Lawrence, 2015b). /k/ has been examined in relation to Liverpool (Watson, 2007b), as has the NURSE-SQUARE merger (Watson and Clark, 2013). school has been examined for Manchester (Baranowski and Turton, 2015; Drummond et al., 2022), and /t/ and cure have been studied in Newcastle (Watt and Milroy, 1999). face and goat have been researched as both Northern English and Sheffield phenomena (Lawrence, 2015a; Finnegan, 2015), and happY has been examined in relation to Sheffield English (Stoddart et al., 1999). It should be reassuring to the researchers noted above that they have been examining features that seem to matter for listeners, although the findings for consonants in this research should encourage further work that examines non-vocalic variation in these varieties and others.

#### 4.1.6 Human perception versus predictive models

It should be noted that the features that listeners attended to were not the ones revealed as being used by the random forest clustering algorithms in Strycharczuk et al. (2020). These features, which have previously attracted little academic interest, were notable due to their role in discriminating between samples in the clustering process. As set out in the Introduction, our study was designed as a direct test of whether these ‘algorithmically salient’ features are also perceptually salient for human listeners. The contrast we observe here therefore speaks directly to that aim, showing that while predictive models and human perception both identify systematic cues, they do not necessarily converge on the same linguistic variables. Our respondents, of course, were undertaking a quite different task. We asked human listeners to specifically spot features as they attempted to work out where a speaker was from. Strycharczuk et al.’s (2020) research did not. It was instead asking a machine learning algorithm to differentiate between vowels from numerous speakers from different locations and sort them into the most likely location. Our listeners were also dealing with a much richer signal, including consonantal and vocalic segmental information as well as the prosodic patterns common to natural speech. Given these factors it is perhaps unsurprising that our respondents did not attend to these lesser-studied features.

### 4.2 Further lines of research

Our research opens possibilities for new routes of enquiry, as well as introducing new questions that could help to understand some of the patterns in our data more fully. Our speakers were all female, young, and from an online corpus of recordings. As a result, the speakers were not only more likely to exhibit less regionally marked speech but will also have encountered listener expectations about regionality in relation to their speech (in the sense that for some listeners the concept of regional speech may also be tied up with concepts of masculinity). Rerunning the experiment with male speakers, as well as older and more regionally marked speakers is therefore something that could be useful.

More speakers could also be used as the use of only one speaker per location could be considered a limitation of the present research. If examining feature attention among many speakers across varieties as in this research, ways to mitigate listener fatigue would need to be addressed. Alternatively, a focus could instead be on single varieties, with multiple samples of speakers from the same location played to listeners. This would permit examination of the extent to which accents are recognized based on the same individual or combined features across speakers.

Although we consider the richness of the stimuli to which respondents were exposed (i.e., one that was unmanipulated and relatively long) to be a strength of the research, it is possible that this richness contributed to a lack of useable data for the analysis we have presented here. As we note above, only 20.84% of the click data provided by listeners was used in this paper because respondents had the opportunity to click and comment on more than segmental features, as well as possessing a limited vocabulary in which to accurately define what they had attended to. For this reason, further research could collect data from a greater number of listeners to provide a larger amount of data. It should also be noted that other analyses can be conducted on the same dataset which permit a greater subset of the click and comment data to be used (such as analyses that examine the strategies listeners use when justifying their clicks).

It would also be useful to use the results from this research in more targeted experimental work, controlling for the presentation of specific variants in shorter stretches of speech, and perhaps adding the lesser-studied features from Strycharczuk et al. (2020) to examine their effect. This type of research could be undertaken in an explicit framework using SLIC or an implicit framework, perhaps including an evaluative component, and would reveal valuable data on the ways in which listeners process accented speech. More targeted investigations, particularly those using the features identified in this paper in manipulated samples, would increase the amount of usable data and allow us to address more directly which features shape listener perceptions. At the same time, the reliance on predictive models such as random forests carries limitations that warrant consideration. These models optimize for statistical classification accuracy, which can privilege highly prototypical or homogeneous tokens and downplay natural within- and between-speaker variability. As a result, model-selected stimuli may not fully capture the range of forms that listeners encounter in everyday speech, and they may highlight cues that matter acoustically but are not those listeners themselves prioritize. Future research should therefore triangulate model-based feature selection with targeted perceptual testing, ensuring that computational predictions are grounded in the social and perceptual realities of accent recognition.

## 5 Conclusion

The research has demonstrated the link between feature attention and different types of salience. It has found variable ability amongst listeners to locate speakers of different Northern English varieties and shown how the specific features present in speaker guises can affect listener perceptions of speaker provenance. We have presented a new approach to the study of listener attention to speech, which showed that the features real listeners notice in five Northern varieties of English largely align with those investigated in sociolinguistics research on the North of England, and corroborate the selection of these features in sociolinguistic research. By setting up SLIC as a direct test of the algorithmic predictions of Strycharczuk et al. (2020), we show that human perception aligns more closely with sociolinguists’ long-standing feature choices than with the machine-selected cues. This suggests that while computational models highlight potentially overlooked variables, they may not map straightforwardly onto what listeners themselves treat as socially or indexically meaningful.

Furthermore, we have shown that accent recognition within the North of England is highest for the two varieties with more distinctive features: English from Newcastle and Liverpool. Recognition was much less accurate for the three central cities: Manchester, Leeds, and Sheffield. Finally, we have argued that social salience should be considered gradable in the way that cognitive salience is.

## Data Availability

The original contributions presented in the study are included in the article/Supplementary material, further inquiries can be directed to the corresponding author.

## Footnotes

^1^The figures for Education and Mobility are as follows: Higher Education (BA, BSc, MA etc., PGCE) & professional/vocational equivalents = 54; A levels, Bac, vocational level 3 etc. = 32; 5 GCSEs grade A*–C, 5 O-Levels, vocational level 2 etc. = 5; Fewer than 5 GCSEs grade A*–C, or fewer than 5 O-Levels = 6; I still live in the same home as 10 years ago = 44; Once = 23; 2–3 times = 18; 4 times or more = 13.
